# Trifluoromethyl-Substituted Large Band-Gap Polytriphenylamines for Polymer Solar Cells with High Open-Circuit Voltages

**DOI:** 10.3390/polym10010052

**Published:** 2018-01-08

**Authors:** Shuwang Yi, Wanyuan Deng, Sheng Sun, Linfeng Lan, Zhicai He, Wei Yang, Bin Zhang

**Affiliations:** 1 State Key Laboratory of Luminescent Materials and Devices, Institute of Polymer Optoelectronic Materials and Devices, South China University of Technology, Guangzhou 510640, China; shuwangyi399@163.com (S.Y.); dwyscut2017@163.com (W.D.); sun.sheng@mail.scut.edu.cn (S.S.); lanlinfeng@scut.edu.cn (L.L.); pswyang@scut.edu.cn (W.Y.); 2 Jiangsu Key Laboratory of Environmentally Friendly Polymeric Materials, School of Materials Science & Engineering, National Experimental Demonstration Center for Materials Science and Engineering, Changzhou University, Changzhou 213164, China

**Keywords:** triphenylamine, trifluoromethyl, large band gap, polymer solar cells, high open circuit voltage

## Abstract

Two large band-gap polymers (**PTPACF** and **PTPA2CF**) based on polytriphenylamine derivatives with the introduction of electron-withdrawing trifluoromethyl groups were designed and prepared by Suzuki polycondensation reaction. The chemical structures, thermal, optical and electrochemical properties were characterized in detail. From the UV-visible absorption spectra, the **PTPACF** and **PTPA2CF** showed the optical band gaps of 2.01 and 2.07 eV, respectively. The cyclic voltammetry (CV) measurement displayed the deep highest occupied molecular orbital (HOMO) energy levels of −5.33 and −5.38 eV for **PTPACF** and **PTPA2CF**, respectively. The hole mobilities, determined by field-effect transistor characterization, were 2.5 × 10^−3^ and 1.1 × 10^−3^ cm^2^ V^−1^ S^−1^ for **PTPACF** and **PTPA2CF**, respectively. The polymer solar cells (PSCs) were tested under the conventional device structure of ITO/PEDOT:PSS/polymer:PC_71_BM/PFN/Al. All of the PSCs showed the high open circuit voltages (*V*_oc_s) with the values approaching 1 V. The **PTPACF** and **PTPA2CF** based PSCs gave the power conversion efficiencies (PCEs) of 3.24% and 2.40%, respectively. Hence, it is a reliable methodology to develop high-performance large band-gap polymer donors with high *V*_oc_s through the feasible side-chain modification.

## 1. Introduction

In the past decades, the photovoltaic cells are one of versatile technologies realizing the clean energy, including the silicon-based inorganic solar cells and carbon-based organic/polymer solar cells [1,2,3]. Among them, bulk-heterojunction polymer solar cells (BHJ PSCs) are becoming an attractive technology both academically and commercially, due to their excellent merits in low cost, flexibility, large-scale fabrication and feasible accessibility of organic chemicals [4,5,6]. Recently, the power conversion efficiencies (PCEs) of BHJ PSCs have been exceeding 14% for the single-junction PSCs [7]. This achievement makes the potential realization in converting the solar power to the electricity for the general marketing application.

In BHJ PSCs, they comprise of a ‘sandwich structure’ which means the BHJ active layer is sandwiched by the anode and cathode, respectively [8]. To achieve the high-performance PSCs, many works are located at the development of active layers containing polymer donors, fullerene and non-fullerene acceptors [9,10,11]. To date, a plenty of polymer donors have been designed and synthesized, such as polycarbazoles [12], polyfluorenes [13], polyindolcarbazoles [14] and polydibenzothiophene (PDBTs) [15,16] derivatives. Among them, a PDBT derivative, PBT7-Th, showed the very high photovoltaic properties in fullerene-based PSCs with the highest PCE over 10% [17]. Due to the weakness of fullerene derivatives in weak light absorption and low stability in PSCs, a series of non-fullerene organic acceptors are developed, such as ITIC [18] and IDIC [19]. The appearance of non-fullerene acceptors stimulates the fast development of large-band gap polymers, resulting from the complementary absorption between polymer donors and non-fullerene acceptors, and matched energy-level alignment [20,21,22]. For instance, a large band-gap polymer poly[(2,6-(4,8-bis(5-(2-ethylhexyl)thiophen-2-yl)-benzo[1,2-b:4,5-b′]dithiophene))-alt-(5,5-(1′,3′-di-2-thienyl-5′,7′-bis(2-ethylhexyl)benzo[1′,2′-c:4′,5′-c′] dithiophene-4,8-dione)] (PBDB-T) could push the PCE to the over 12% by using the IT-M as the electron acceptor [23]. Therefore, these successful results open the new era for synthetic chemistry and device engineering in PSCs.

**Figure 1:**
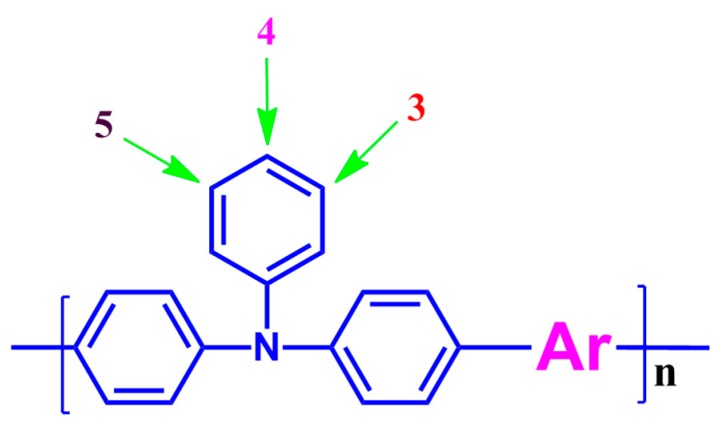
Chemical structure of triphenylamine-based conjugated polymer.

Triphenylamine (TPA) is a versatile aromatic moiety which is utilized widely in organic electronics, attributing to its advantages in low cost, feasibly chemical modification, high hole mobility, highly thermal stability and deep highest occupied molecular orbital (HOMO) energy levels [24,25]. The functional molecules prepared from TPA showed high hole transporting ability and high light-emitting properties in organic light-emitting diodes (OLEDs) [26]. Furthermore, the TPA-based conjugated polymers or small molecules can be utilized as electron donors and electron acceptors in PCSs [27,28]. Due to the deep HOMO energy level of TPA unit, the polymers based on TPA also displayed the high open circuit voltages (*V*_oc_s) in PSCs [29]. Moreover, the nature of high hole mobility triggers the huge application of TPA-based molecules and polymers as hole transporting layers (HTLs) in organic-halide perovskite solar cells (PVSCs); owing to the introduction of these versatile materials in PVSCs, both of the *V*_oc_s and film formability were improved dramatically with the very high photovoltaic performance [30]. Hence, these achievements of TPA-based molecules and polymers in optoelectronic devices will continue the drastic developments of new TPA-based materials via synthetic functionalization. As displayed in Figure 1, the bare TPA unit has the potential modification by anchoring functional groups to improve the optoelectronic properties. For instance, the bare positions of 3-, 4- and 5- are un-functionalized, and they could be modified by introducing the electron-withdrawing or electron-donating groups to tune the energy levels of the targeting polymers.

Herein, two large band-gap TPA-based polymers were designed and synthesized. One of them is trifluoromethyl-substituted polymer (**PTPACF**), and another is bis(trifluoromethyl)-substituted polymer (**PTPA2CF**). Ascribing to the electron-withdrawing ability of trifluoromethyl group, the HOMO energy levels of **PTPACF** and **PTPA2CF** can be tuned to deeper level approaching to about −5.40 eV. Through testing the photovoltaic performance using **PTPACF** and **PTPA2CF** as polymer donors and [6,6]-phenyl-C71-butyric acid methyl ester (PC_71_BM) as the acceptors, the *V*_oc_s were improved to about 1 V. Therefore, this is a reasonable methodology to develop the high-performance polymer donors with high *V*_oc_ values.

## 2. Experimental Section

### 2.1. Characterization and Instrumentation

Through utilizing Bruker 300 (or 600) MHz DRX spectrometer (Bruker, Karlsruhe, Germany) with tetramethylsilane (TMS) as the reference, the ^1^H nuclear magnetic resonance (NMR) and ^13^C NMR spectra were characterized in detail. By using the linear polystyrene (PS) as standards with the tetrahydrofuran (THF) as eluent, the number-averaged molecular weight (*M*_n_) and weight-averaged molecular weight (*M*_w_) of the targeting polymers were tested on a Waters gel permeation chromatography (GPC) system. Cyclic voltammetry (CV) measurement was performed on a CHI600D electrochemical workstation (CH Instruments, Shanghai, China) in a nitrogen-saturated anhydrous acetonitrile solution with a 0.1 mol·L^-1^ tetrabutylammonium hexafluorophosphates (Bu4NPF6) as electrolyte. In CV test, it is utilized with a standard three electrodes cell, comprising of a platinum (Pt) working electrode, a Pt wire counter electrode, against saturated calomel electrode (SCE) as reference electrode. At the same time, the scan rate was performed at 50 mV·s^-1^, and the ferrocene/ferrocenium (Fc/Fc^+^) was applied as the internal reference. The results of UV-vis absorption spectra were recorded on a SHIMADZU UV-3600 spectrophotometer (SHIMADZU, Kyoto, Japan). The atomic force microscopy (AFM) under the tapping-mode was carried out on a Veeco Nanoscope V scanning probe microscope, for testing topography and phase images of blending films. 

### 2.2. Device Fabrication

All devices were fabricated on indium tin oxide (ITO)-coated glass substrates. A thin-layer (40 nm) of poly(3,4-ethylenedioxythiophene):poly(4-styrenesulfonate) (PEDOT:PSS) (Clevios 4083) was spin-coated onto UV-ozone treated ITO substrates at 4000 rpm for 40 s and then baked at 140 °C for 15 min in air. The solution containing the polymers and PC_71_BM with different weight ratios in *o*-dichlorobenzene (ODCB) were spin-coated inside a glove-box with nitrogen at 1200 rpm for 40 s to form the active layers (~80 nm). In this study, the conventional device structure was ITO/PEDOT-PSS/active layer/ PFN/Al, where the PFN is poly[(9,9-dioctyl-2,7-fluorene)-alt-(9,9-bis (3′-(*N*,*N*-dimethylamino)propyl)-2,7-fluorene)]. The PFN layer with a thickness of about 5 nm was prepared by spin-casting a mixed solution (0.2 mg/ml) in methanol with a trace of acetic acid before the cathode evaporation. Aluminum (80 nm) was utilized as cathode by thermally evaporation under vacuum (~10^−6^ torr) with a shadow mask of 0.16 cm^2^. The current-voltage (*J*-*V*) curves were measured on a Keithley 2400 multimeter under AM 1.5 G solar illumination at 100 mW cm^−2^. The incident photon-to-current conversion efficiency (IPCE) values were recorded by a monochromator under calibrating with a silicon photodiode. Hole mobility data of neat polymer films were measured by field-effect transistors (FETs) method with the device configuration of Al/AlOx:Nd/PMMA/**PTPACF** or **PTPA2CF**/Au (*W*/*L* = 500 μm/70 μm).

### 2.3. Synthesis of Monomers and Polymers

All the starting materials and reagents were utilized by purchasing commercially without further purification. [6,6]-phenyl-C71-butyric acid methyl ester (PC_71_BM) was purchased from Lumtec Corp. (Taipei, Taiwan). The monomers of 4,7-Bis(5-bromothiophen-2-yl)-5,6-bis (octyloxy)benzothiadizole **1**, *N*,*N*-bis(4-(4,4,5,5-tetramethyl-1,3,2-dioxaborolan-2-yl)phenyl)-4-(trifluoromethyl)phenyl)aniline **2** (283 mg, 0.5 mmol) or *N*,*N*-bis(4-(4,4,5,5-tetramethyl-1,3,2-dioxaborolan-2-yl)phenyl)-3,5-bis(trifluoromethyl)aniline **3**, were synthesized according to the published literatures [29]. The targeting polymers in this study were prepared by the following synthetic methodologies.

#### 2.3.1. General Procedure for Preparing Polymers

*N*,*N*-bis(4-(4,4,5,5-tetramethyl-1,3,2-dioxaborolan-2-yl)phenyl)-4-(trifluoromethyl)phenyl)aniline **2** (283 mg, 0.5 mmol) or *N*,*N*-bis(4-(4,4,5,5-tetramethyl-1,3,2-dioxaborolan-2-yl)phenyl)-3,5-bis(trifluoromethyl)aniline **3** (317 mg, 0.5 mmol), 4,7-Bis(5-bromothiophen-2-yl)-5,6-bis (octyloxy) benzothiadizole **1** (349 mg, 0.5 mmol) and Pd(PPh_3_)_4_ (11.6 mg, 0.01 mmol) were dissolved in 10 mL toluene under the nitrogen protection. The organic basic tetraethyl ammonium hydroxide aqueous solution (2 mL, 20 wt/wt %) was added in one portion and the mixture was heated to 80 °C and continued to react for 48 h. After 48 h, phenylboronic acid (0.609 g, 5 mmol) in THF was added and reacted for additional 12 h; then, bromobenzene (1.57 g, 10 mmol) was injected into the reaction system in one portion and reacted for another 12 h. After that, the mixture was cooled to room temperature and precipitated in methanol. The crude polymers were then purified by Soxhlet extraction method in methanol, acetone, hexane and chloroform respectively. Finally, the chloroform solution containing the polymers was precipitated in methanol, and filtered off and dried at 60 °C in the vacuum for 10 h.

#### 2.3.2. Poly(*N,N*-diphenyl-4-(trifluoromethyl)aniline-alt-4,7di(thiophen-2-yl)-5,6-bis(octyloxy)benzothiadiazole) **PTPACF**

Yielding 215 mg of the target polymer as a red solid. ^1^H NMR (CDCl_3_, 600 MHz, δ): 8.29–8.12 (m, 2H, Ar–H), 7.57–7.30 (br, 8H, Ar–H), 7.13–6.95 (br, 6H, Ar–H), 4.13–4.07 (m, 4H, CH2), 1.98–1.93 (m, 4H, CH2), 1.42–1.17 (br, 20H, CH2), 0.89–0.76 (m, 6H, CH3). GPC (THF): *M*_n_ = 9100, *M*_w_ = 15700, PDI = 1.73.

#### 2.3.3. Poly(*N,N*-diphenyl-3,5-bis(trifluoromethyl)aniline-alt-4,7di(thiophen-2-yl)-5,6-bis(octyloxy)benzothiadiazole) **PTPA2CF**

Yielding 175 mg of the target polymer as a red solid. ^1^H NMR (CDCl_3_, 600 MHz, δ): 8.62 (s, 1H, Ar–H), 7.65–7.27 (br, 10H, Ar–H), 7.18–7.12 (br, 4H, Ar–H), 4.21–4.15 (m, 4H, CH2), 2.05–2.02 (m, 4H, CH2), 1.51–1.24 (br, 20H, CH2), 0.93–0.86 (m, 6H, CH3). GPC (THF): *M*_n_ = 8400, *M*_w_ = 12900, PDI = 1.54.

## 3. Results and Discussion

### 3.1. Synthesis

**Scheme 1:**
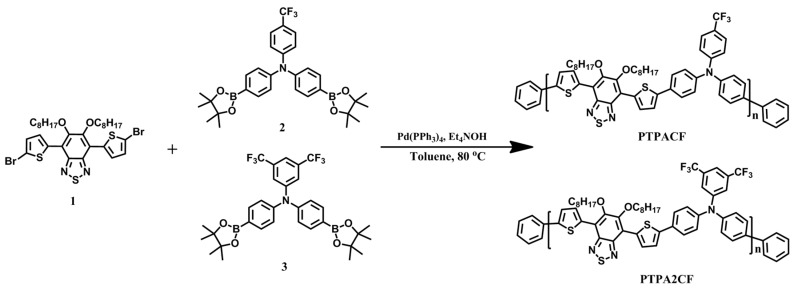
The synthetic routes for polymers.

The detailed synthetic routes of polymers are presented in Scheme 1. The polymers **PTPACF** and **PTPA2CF** were prepared via the general Suzuki polycondensation in the presence of active Pd(PPh_3_)_4_ as the catalyst. The crude polymers were purified via the Soxhlet extraction by methanol, acetone, hexane and chloroform, respectively. Finally, the chloroform solution containing the polymers was precipitated in methanol to obtain the targeting polymers as the red solids. The **PTPACF** and **PTPA2CF** showed high solubility in common organic solvents, such as toluene, tetrahydrofuran, chloroform and chlorobenzene. The molecular weights of **PTPACF** and **PTPA2CF** were characterized by the Gel Permeation Chromatography (GPC), using the tetrahydrofuran as the eluent. The measured number-averaged molecular weights (*M*_n_s) of **PTPACF** and **PTPA2CF** were 9100 and 8400 with the polydispersity index (PDIs) of 1.73 and 1.54, respectively. (See Table 1)

### 3.2. Thermal Stability

**Figure 2:**
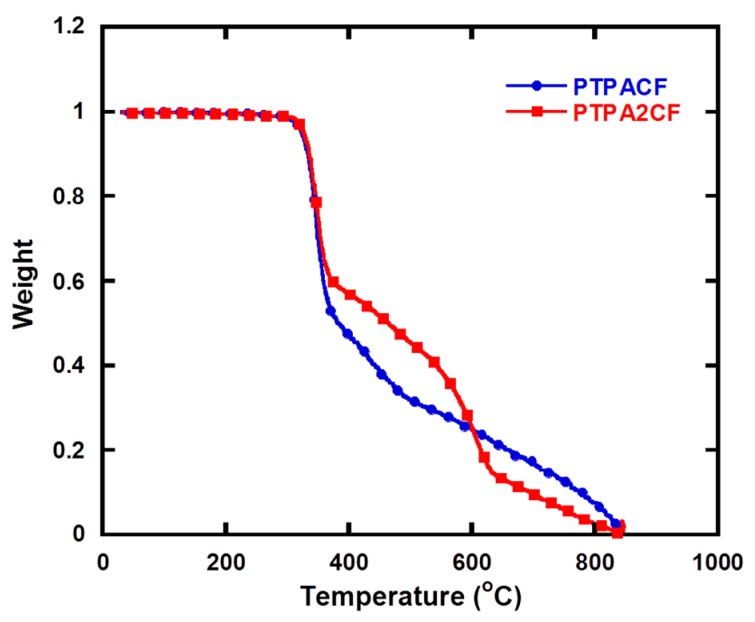
Thermal gravimetric analysis curves of the PTPACF and PTPA2CF.

In PSCs, the thermal stability of polymers is one of the significant parameters to evaluate the device sustainability and life time, and the highly thermal stability of polymers will permit the high stability of PSCs for longtime application [31,32,33]. In this study, the thermal gravimetric analysis (TGA) was characterized to estimate the thermal stability of **PTPACF** and **PTPA2CF**, and the corresponding TGA curves are presented in Figure 2. From the TGA curves, it is found that the degradation temperatures at 5% weight loss of **PTPACF** and **PTPA2CF** are 335 and 338 °C, respectively. (See Table 1) These high degradation temperatures indicate that all of the polymers can be stable up to 300 °C. 

### 3.3. UV-vis Absorption and Electrochemical Properties

**Figure 3:**
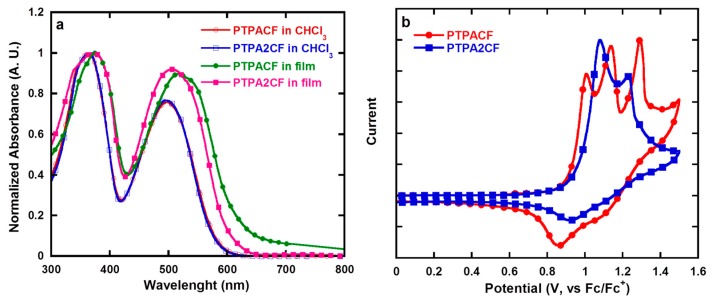
UV-vis absorption spectra (**a**) and cyclic voltammogram curves (**b**) of the polymers.

The UV-vis absorption spectra of the polymers in chloroform and in thin film are shown in Figure 3a. The details acquired from UV-vis absorption measurement are summarized in Table 2. From Figure 3a, these two polymers display the classic “double hump” absorption peaks; some are located at short wavelength due to the localized π–π* transition and the others appear at long wavelength resulting from the internal charge transfer (ICT) interaction between the donor and acceptor units [34]. In chloroform solution, **PTPACF** and **PTPA2CF** showed the very similar absorption spectra around 363 and 495 nm because of the similarly chemical structures. In solid thin films, both of **PTPACF** and **PTPA2CF** give the red-shifted absorption spectra by more than 10 nm, which indicate that they form the π–π stacking between the polymer main chains in the solid state. The onsets of the UV-vis absorption spectra in solid films are 616 and 610 nm, corresponding to the optical band gaps of 2.01 and 2.07 eV for **PTPACF** and **PTPA2CF**, respectively [35]. From optical band gaps, it implies that all of these two polymers present the large band gaps.

The HOMO energy levels (*E*_HOMO_s) of polymers were demonstrated by cyclic voltammetry (CV) measurement. Electrochemical CV characterization were carried out by the well-known method with the three electrodes of Pt electrode, saturated calomel electrode, and Pt wire utilized as the working electrode, reference electrode, and counter electrode, respectively, and 0.1 mol·L^−1^ tetrabutylammonium hexafluorophosphate (Bu_4_NPF_6_) solution in dry acetonitrile (CH_3_CN) was used as the electrolyte. The E_HOMO_s of the polymers were calculated from CV measurements using the oxidation onset potentials relative to ferrocene as an internal standard, and the relative CV curves are shown in Figure 3b. The redox potential of Fc/Fc^+^ internal reference is 0.39 V vs. SCE. The E_HOMO_s of **PTPACF** and **PTPA2CF**, determined by calculating from the empirical formula of *E*_HOMO_ = −e(*E*_ox_ + 4.8 − *E*_1/2, (Fc/Fc+)_), are −5.33 and −5.38 eV, respectively [36]. As we did not obtain the reductive curves from CV measurements, the lowest unoccupied molecular orbit (LUMO) energy levels (*E*_LUMO_s) of polymers were determined by calculating from the formula of *E*_LUMO_ = *E*_g, optical_ + *E*_HOMO_, where *E*_g, optical_ means the optical band gap. Through the calculation, *E*_LUMO_s of **PTPACF** and **PTPA2CF** are −3.32 and −3.31 eV, respectively. From CV data, we can see that both of these two polymers show the deep HOMO energy levels, which will potentially realize the high *V*_oc_s in these two polymers based PSCs.

### 3.4. Hole Mobility

**Figure 4:**
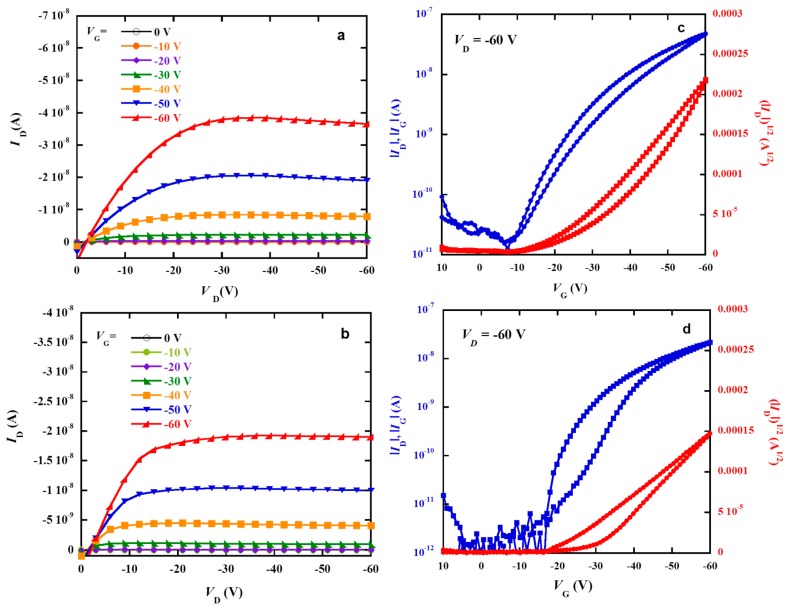
Output (**a**,**b**) and transfer (**c**,**d**) characteristics of the PTPACF (**a**,**c**) and PTPA2CF (**b**,**d**) based OFETs under the device structure of Al/AlOx:Nd/PMMA/polymer/Au (*W*/*L* = 500/70 μm).

To evaluate the charge transporting properties of semiconducting polymers, the hole mobility is an important parameter for polymer donors in PSCs. In this study, we acquired the hole mobility data of **PTPACF** and **PTPA2CF** by utilizing the field-effect transistors (FETs) method. The detailed FETs properties containing output and transfer characteristics of the **PTPACF** and **PTPA2CF** under the device structure of Al/AlOx:Nd/PMMA/**PTPACF** or **PTPA2CF** /Au (*W*/*L* = 500 μm/70 μm) were displayed in Figure 4, and the hole mobility values were summarized in Table 3. From the FETs characterization, it shows that the hole mobilities are 2.5 ×10^−3^ and 1.1 × 10^−3^ cm^2^·V^−1^·S^−1^ for **PTPACF** and **PTPA2CF**, respectively. As a comparison, we can see that the hole mobilities of **PTPACF** and **PTPA2CF** coincide with the results based on the TPA-derived polymers published in the literature, in which the hole mobilities would be affected efficiently by different modification of substituting groups and co-polymerizing monomers [27,29,37]. Furthermore, the **PTPACF** gives the twice higher hole mobility than **PTPA2CF**, which is possibly because **PTPACF** exists in a better molecular stacking state than **PTPA2CF**. These high hole mobilities will be potentially beneficial for the charge transport in PSCs and achieving high photovoltaic performance.

### 3.5. Photovoltaic Performance

**Figure 5:**
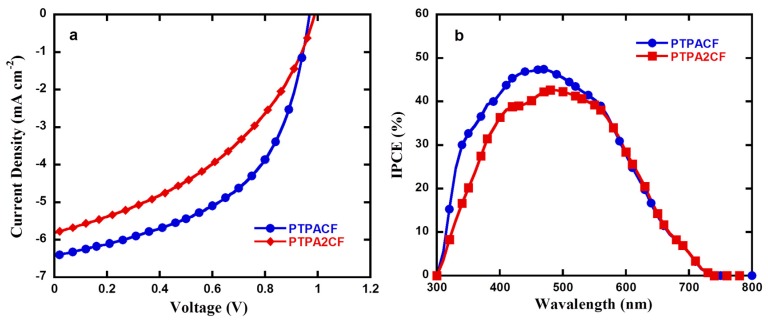
*J*–*V* characteristics (**a**) and the corresponding IPCE spectra (**b**) of PTPACF:PC_71_BM and PTPA2CF:PC_71_BM based PSCs, under the illumination of AM1.5G, 100 mW·cm^−2^.

To study the photovoltaic performance of these two polymers as donors, the PSCs based on **PTPACF** and **PTPA2CF** were fabricated under the device structure of ITO/PEDOT:PSS/polymer: PC_71_BM/PFN/Al, where the PFN is poly[(9,9-dioctyl-2,7-fluorene)-alt-(9,9-bis(30-(*N*,*N*-dimethylamino)propyl)-2,7-fluorene)]. The optimized weight ratio of polymer to PC_71_BM is 1:4. Here, the PSCs were prepared without further post treatment, such as thermal annealing and solvent additives. Moreover, PFN was utilized as a cathode interfacial layer in this device architecture attributing to its versatile functions in protecting active layer when cathode evaporation, and decreasing the work function of cathode and possibly existing the built-in electric fields between the active layer and cathode [38,39,40]. The current density-voltage (*J*-*V*) curves and incident photon-to-current conversion efficiency (IPCE) curves measured under AM1.5G, 100 mW·cm^−2^ are shown in Figure 5, and the photovoltaic properties in details are summarized in Table 3. From the photovoltaic characterization, it was found that all devices incorporating these two polymers showed short circuit current density (*J*_sc_) at about 6 mA cm^−2^, but they gave the quite impressive high *V*_oc_ values of about 1.0 V resulting from the deep HOMO energy levels of **PTPACF** and **PTPA2CF** within the modification by electron-withdrawing trifluoromethyl groups. Both of **PTPACF** and **PTPA2CF** shows the higher *V*_oc_s than the other published bare TPA-based polymers without any substitution. In these bare TPA-based PSCs, the values of *V*_oc_ were only located at about 0.9 V [27,29]. The **PTPACF**-based PSC showed the *J*_sc_ of 6.42 mA·cm^−2^, *V*_oc_ of 0.98 V, fill factor (FF) of 52.0% and PCE of 3.24%, respectively. On the other hand, the **PTPA2CF**-based PSC displayed the *J*_sc_ of 5.81 mA·cm^−2^, *V*_oc_ of 0.99 V, FF of 41.8% and PCE of 2.40%, respectively. From photovoltaic performance, **PTPA2CF** gives the higher *V*_oc_ than **PTPACF**-based PSC, which is possibly because the **PTPA2CF** has the deeper HOMO energy level than **PTPACF**. This deeper HOMO energy level may improve the *V*_oc_ positively in **PTPA2CF**-based PSC. Furthermore, the high hole mobility of **PTPACF** also improve the charge carriers transport and helps to explain the high *J*_sc_ and FF achieved in **PTPACF**:PC_71_BM-based device. The corresponding IPCE curves in Figure 5b exhibited a broad photoresponse in the range of 300–700 nm for all polymers-based PSCs. Especially for **PTPACF**:PC_71_BM device, IPCE exceeded 30% from 330 to 580 nm, with a peak of 48% at 470 nm. Therefore, **PTPACF** based device yielded the highest *J*_sc_. The calculated *J*_sc_s from the IPCE spectra are in good agreement with the values obtained from *J*-*V* measurements.

### 3.6. Atomic Force Microscopy (AFM) Morphologies

**Figure 6:**
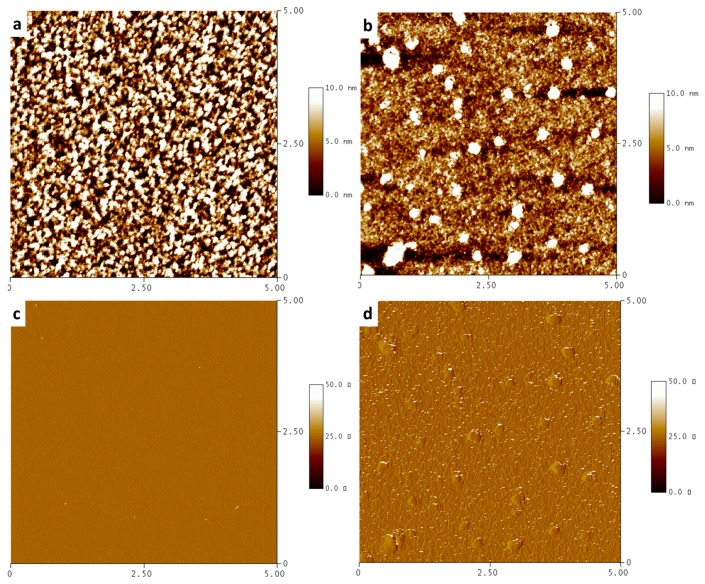
AFM topography images (5 μm × 5 μm) of (**a**) **PTPACF**:PC_71_BM blend film and (**b**) **PTPA2CF**:PC_71_BM blend film; AFM phase images of (**c**) **PTPACF**:PC_71_BM blend film and (**d**) **PTPA2CF**:PC_71_BM blend film.

To study the interaction of blend films between these two polymers and PC_71_BM, the Atomic Force Microscope (AFM) images of the blend films are shown in Figure 6. In the topography of Figure 6a,b, the root-mean-square (RMS) roughness of blend films are 0.476, 0.23 nm for **PTPACF** and **PTPA2CF**, respectively, which means that **PTPACF** and **PTPA2CF** based blend films have quite smoothly topographic morphologies with less obvious density fluctuations. Furthermore, the phase images (Figure 6c,d) display that **PTPACF**-based blend films give the better nano-scale phase separation, while **PTPA2CF**-based blend films show worse phase separation and form bigger aggregation that would cause carrier transport limitations and nongeminate recombination seriously, and ultimately decrease the FF values and photovoltaic performance. According to the photovoltaic performance in Table 3, it also depicts that **PTPACF** exhibits higher FF value than **PTPA2CF**, ascribing to their better nano-scale phase separation in **PTPACF**:PC_71_BM blend film.

## 4. Conclusions

In summary, two large band-gap polymer donors **PTPACF** and **PTPA2CF** with different trifluoromethyl substitution in TPA unit were designed and synthesized. The optical band gaps of **PTPACF** and **PTPA2CF** were 2.01 and 2.07 eV, respectively. Ascribing to the introduction of electron-withdrawing trifluoromethyl groups, the **PTPACF** and **PTPA2CF** showed deep HOMO energy levels with −5.33 and −5.38 eV, respectively. Through the photovoltaic characterization in BHJ PSCs, these two polymer donors displayed high *V*_oc_s approaching to 1 V. These results with high *V*_oc_ values are very beneficial for the PSCs realizing highly photovoltaic performance. Even though the PCEs of **PTPACF** and **PTPA2CF** based PSCs by utilizing PC_71_BM as electron acceptors are not so high, it may be believed that the higher photovoltaic performance would be achieved in non-fullerene PSCs by using the low band-gap organic acceptors, originating from the versatile merits of **PTPACF** and **PTPA2CF** in large band gaps and low HOMO energy levels. Hence, it is a feasibly accessible way to develop high-performance polymer donors via side-chain functionalization in polytriphenylamine derivatives.

## Figures and Tables

**Table 1:** Molecular weights and thermal stability for the polymers.

Polymers	*M*_n_	*M*_w_	PDI	*T*_d_ (°C)
**PTPACF**	9100	15,700	1.73	335
**PTPA2CF**	8400	12,900	1.54	338

**Table 2:** Summary of UV-vis absorption and electrochemical properties.

Polymers	λ_max,_ _in chloroform_ (nm)	λ_max,_ _in film_ (nm)	λ_onset_ (nm)	*E*_g, optical_ (eV)	*E*_ox_ (V)	*E*_HOMO_ (eV)	*E*_LU__MO_ (eV)
**PTPACF**	363, 498	375, 516	616	2.01	0.92	−5.33	−3.32
**PTPA2CF**	365, 495	373, 505	600	2.07	0.97	−5.38	−3.31

**Table 3:** Summarized photovoltaic performance of the PTPACF:PC_71_BM and PTPA2CF:PC_71_BM based PSCs under the illumination of AM1.5G, 100 mW cm^−2^.

Polymers	μ (×10^−3^, cm^2^ V^−1^ S^−1^)	*J*_sc_ (mA cm^−2^)	*V*_oc_ (V)	FF (%)	PCE (%)
**PTPACF**	2.5	6.42	0.98	52.0	3.24
**PTPA2CF**	1.1	5.81	0.99	41.8	2.40

## References

[1] Sivakov V., Andrä G., Gawlik A., Berger A., Plentz J., Falk F., Christiansen S.H. (2009). Silicon nanowire-based solar cells on glass: Synthesis, optical properties, and cell parameters. Nano Lett..

[2] Dou L., You J., Hong Z., Xu Z., Li G., Street R.A., Yang Y. (2013). 25th anniversary article: A decade of organic/polymeric photovoltaic research. Adv. Mater..

[3] Cheng Y., Yang S., Hsu C. (2009). Synthesis of conjugated polymers for organic solar cell applications. Chem. Rev..

[4] Li G., Zhu R., Yang Y. (2012). Polymer solar cells. Nat. Photonics.

[5] Helgesen M., Søndergaard R., Krebs F.C. (2010). Advanced materials and processes for polymer solar cell devices. J. Mater. Chem..

[6] Wong W., Ho C. (2010). Organometallic photovoltaics: A new and versatile approach for harvesting solar energy using conjugated polymetallaynes. Acc. Chem. Res..

[7] Xiao Z., Jia X., Ding L. (2017). Ternary organic solar cells offer 14% power conversion efficiency. Sci. Bull..

[8] Dennler G., Scharber M.C., Brabec C.J. (2009). Polymer-fullerene bulk-heterojunction solar cells. Adv. Mater..

[9] Soci C., Hwang I.W., Moses D., Zhu Z., Waller D., Gaudiana R., Brabec C.J., Heeger A.J. (2007). Photoconductivity of a low-bandgap conjugated polymer. Adv. Funct. Mater..

[10] Wang Q., Li Y., Song P., Su R., Ma F., Yang Y. (2017). Non-fullerene acceptor-based solar cells: From structural design to interface charge separation and charge transport. Polymers.

[11] Gao Y., Liu M., Zhang Y., Liu Z., Yang Y., Zhao L. (2017). Recent development on narrow bandgap conjugated polymers for polymer solar cells. Polymers.

[12] Zhang B., Hu X., Wang M., Xiao H., Gong X., Yang W., Cao Y. (2012). Highly efficient polymer solar cells based on poly(carbazole-alt-thiophene-benzofurazan). New J. Chem..

[13] Guo X., Baumgarten M., Müllen K. (2013). Designing π-conjugated polymers for organic electronics. Prog. Polym. Sci..

[14] Zhang B., Yu L., Fan L., Wang N., Hu L.W., Yang W. (2014). Indolo[3,2-*b*]carbazole and benzofurazan based narrow band-gap polymers for photovoltaic cells. New J. Chem..

[15] Liang Y., Xu Z., Xia J., Tsai S., Wu Y., Li G., Ray C., Yu L. (2010). For the bright future-bulk heterojunction polymer solar cells with power conversion efficiency of 7.4%. Adv. Mater..

[16] Liao S., Jhuo H., Cheng Y., Chen S. (2013). Fullerene derivative-doped zinc oxide nanofilm as the cathode of inverted polymer solar cells with low-bandgap polymer (PTB7-Th) for high performance. Adv. Mater..

[17] He Z., Xiao B., Liu F., Wu H., Yang Y., Xiao S., Wang C., Russell T.P., Cao Y. (2015). Single-junction polymer solar cells with high efficiency and photovoltage. Nat. Photonics.

[18] Lin Y., Wang J., Zhang Z., Bai H., Li Y., Zhu D., Zhan X. (2015). An electron acceptor challenging fullerenes for efficient polymer solar cells. Adv. Mater..

[19] Lin Y., He Q., Zhao F., Huo L., Mai J., Lu X., Su C., Li T., Wang J., Zhu J. (2016). A facile planar fused-ring electron acceptor for as-cast polymer solar cells with 8.71% efficiency. J. Am. Chem. Soc..

[20] Lin Y., Zhao F., Wu Y., Chen K., Xia Y., Li G., Prasad S.K.K., Zhu J., Huo L., Bin H. (2017). Mapping polymer donors toward high-efficiency fullerene free organic solar cells. Adv. Mater..

[21] Bin H., Gao L., Zhang Z., Yang Y., Zhang Y., Zhang C., Chen S., Xue L., Yang C., Xiao M. (2016). 11.4% Efficiency non-fullerene polymer solar cells with trialkylsilyl substituted 2D-conjugated polymer as donor. Nat. Commun..

[22] Zhao W., Qian D., Zhang S., Li S., Inganäs O., Gao F., Hou J. (2016). Fullerene-free polymer solar cells with over 11% efficiency and excellent thermal stability. Adv. Mater..

[23] Li S., Ye L., Zhao W., Zhang S., Mukherjee S., Ade H., Hou J. (2016). Energy-level modulation of small-molecule electron acceptors to achieve over 12% efficiency in polymer solar cells. Adv. Mater..

[24] Roquet S., Cravino A., Leriche P., Alévêque O., Frère P., Roncali J. (2006). Triphenylamine-thienylenevinylene hybrid systems with internal charge transfer as donor materials for heterojunction solar cells. J. Am. Chem. Soc..

[25] Lu Q., Cai W., Niu H., Wang W., Bai X., Hou Y. (2017). Novel polyamides with 5*H*-dibenzo[*b*,*f*]azepin-5-yl-substituted triphenylamine: Synthesis and visible-NIR electrochromic properties. Polymers.

[26] Hancock J.M., Gifford A.P., Zhu Y., Lou Y., Jenekhe S.A. (2006). *n*-Type conjugated oligoquinoline and oligoquinoxaline with triphenylamine endgroups: Efficient ambipolar light emitters for device applications. Chem. Mater..

[27] Zhang B., Liang J., Hu L., Peng F., Chen G., Yang W. (2015). Triphenylamine-based broad band-gap polymers for bulk-heterojunction polymer solar cells. J. Mater. Sci..

[28] Luo Z., Xiong W., Liu T., Cheng W., Wu K., Sun Y., Yang C. (2017). Triphenylamine-cored star-shape compounds as non-fullerene acceptor for high-efficiency organic solar cells: Tuning the optoelectronic properties by S/Se-annulated perylene diimide. Org. Electron..

[29] Zhang B., Chen G., Xu J., Hu L., Yang W. (2016). Feasible energy level tuning in polymer solar cells based on broad band-gap polytriphenylamine derivatives. New J. Chem..

[30] Chen G., Zhang F., Liu M., Song J., Lian J., Zeng P., Yip H., Yang W., Zhang B., Cao Y. (2017). Fabrication of high-performance and low-hysteresis lead halide perovskite solar cells by utilizing a versatile alcohol-soluble bispyridinium salt as an efficient cathode modifier. J. Mater. Chem. A.

[31] Griffini G., Douglas J.D., Piliego C., Holcombe T.W., Turri S., Fréchet J.M.J., Mynar J.L. (2011). Long-term thermal stability of high-efficiency polymer solar cells based on photocrosslinkable donor-acceptor conjugated polymers. Adv. Mater..

[32] Müller C. (2015). On the glass transition of polymer semiconductors and its impact on polymer solar cell stability. Chem. Mater..

[33] Holmes N.P., Vaughan B., Williams E.L., Kroon R., Anderrson M.R., Kilcoyne A.L.D., Sonar P., Zhou X.J., Dastoor P.C., Belcher W.J. (2017). Diketopyrrolopyrrole-based polymer:fullerene nanoparticle films with thermally stable morphology for organic photovoltaic applications. MRS Commun..

[34] Hedström S., Henriksson P., Wang E., Andersson M.R., Persson P. (2014). Light-harvesting capabilities of low band gap donor-acceptor polymers. Phys. Chem. Chem. Phys..

[35] Surin M., Hennebicq E., Ego C., Marsitzky D., Grimsdale A.C., Müllen K., Brédas J., Lazzaroni R., Leclère P. (2004). Correlation between the microscopic morphology and the solid-state photoluminescence properties in fluorene-based polymers and copolymers. Chem. Mater..

[36] Cardona C.M., Li W., Kaifer A.E., Stockdale D., Bazan G.C. (2011). Electrochemical considerations for determining absolute frontier orbital energy levels of conjugated polymers for solar cell applications. Adv. Mater..

[37] Cravino A., Roquet S., Alévêque O., Leriche P., Frère P., Roncali J. (2006). Triphenylamine-oligothiophene conjugated systems as organic semiconductors for opto-electronics. Chem. Mater..

[38] He Z., Wu H., Cao Y. (2014). Recent advances in polymer solar cells: Realization of high device performance by incorporating water/alcohol-soluble conjugated polymers as electrode buffer layer. Adv. Mater..

[39] Xia R., Leem D., Kirchartz T., Spencer S., Murphy C., He Z., Wu H., Su S., Cao Y., Kim J.S. (2013). Investigation of a conjugated polyelectrolyte interlayer for inverted polymer:fullerene solar cells. Adv. Energy Mater..

[40] He Z., Zhong C., Su S., Xu M., Wu H., Cao Y. (2012). Enhanced power-conversion efficiency in polymer solar cells using an inverted device structure. Nat. Photonics.

